# Fetal and neonatal outcomes of posterior fossa anomalies: a retrospective cohort study

**DOI:** 10.1038/s41598-024-59163-8

**Published:** 2024-04-10

**Authors:** Hanan Alsehli, Saeed Mastour Alshahrani, Shatha Alzahrani, Farouq Ababneh, Nawal Mashni Alharbi, Nassebah Alarfaj, Duaa Baarmah

**Affiliations:** 1 King Saud Bin Abdulaziz University for Health Sciences, Riyadh, Saudi Arabia; 2 Department of Obstetrics and Gynecology, King Abdulaziz Medical City, Ministry of the National Guard-Health Affairs, Riyadh, Saudi Arabia; 3 King Abdullah International Medical Research Center, Riyadh, Saudi Arabia; 4 Department of Public Health, College of Applied Medical Sciences, King Khalid University, Abha, Saudi Arabia; 5 Department of Pediatric Neurology, King Abdullah Specialist Children Hospital, Ministry of the National Guard-Health Affairs, Riyadh, Saudi Arabia; 6 Department of Genetics and Precision Medicine, King Abdullah Specialist Children Hospital, Ministry of the National Guard-Health Affairs, Riyadh, Saudi Arabia; 7 King Abdulaziz Medical City, Ministry of the National Guard-Health Affairs, Riyadh, Saudi Arabia; 8 Department of Pediatrics, King Abdullah Bin Abdulaziz University Hospital, Princess Nourah Bint Abdulrahman University, Riyadh, Saudi Arabia

**Keywords:** Genetics, Medical research, Neurology

## Abstract

The primary aim of this study was to estimate the incidence of posterior fossa anomalies (PFA) and assess the associated outcomes in King Abdulaziz Medical City (KAMC), Riyadh. All fetuses diagnosed by prenatal ultrasound with PFA from 2017 to 2021 in KAMC were analyzed retrospectively. PFA included Dandy–Walker malformation (DWM), mega cisterna magna (MCM), Blake's pouch cyst (BPC), and isolated vermian hypoplasia (VH). The 65 cases of PFA were 41.5% DWM, 46.2% MCM, 10.8% VH, and 1.5% BPC. The annual incidence rates were 2.48, 2.64, 4.41, 8.75, and 1.71 per 1000 anatomy scans for 2017, 2018, 2019, 2020, and 2021, respectively. Infants with DWM appeared to have a higher proportion of associated central nervous system (CNS) abnormalities (70.4% vs. 39.5%; p-value = 0.014) and seizures than others (45% vs. 17.9%; p-value = 0.041). Ten patients with abnormal genetic testing showed a single gene mutation causing CNS abnormalities, including a pathogenic variant in MPL, C5orf42, ISPD, PDHA1, PNPLA8, JAM3, COL18A1, and a variant of uncertain significance in the PNPLA8 gene. Our result showed that the most common PFA is DWM and MCM. The autosomal recessive pathogenic mutation is the major cause of genetic disease in Saudi patients diagnosed with PFA.

## Introduction

Central nervous system (CNS) malformations are the second most common fetal anomalies, following cardiac anomalies. Therefore, prenatal diagnosis is very important because of the high rates of perinatal morbidity and mortality^1^; besides, it would help with counseling and further follow-up. One of the CNS anomalies is posterior fossa anomalies (PFA), which can be associated with hypotonia, developmental delay, microcephaly, or hydrocephalus^2^.

Posterior fossa abnormalities include Dandy–Walker malformation (DWM), mega cisterna magna (MCM), Blake’s pouch cyst (BPC), and isolated vermian hypoplasia (VH)^3^. The spectrum of PFA is found in about 1 in 5000 live births^4^. The variability of the prognosis and neurodevelopmental outcome depends on different factors, including the type of PFA and whether it is isolated PFA or associated with another CNS or extra CNS abnormalities, also if it is associated with chromosomal abnormalities or not^5,6^. Associated CNS anomalies include ventriculomegaly, hydrocephalus, and agenesis of the corpus callosum. Extra CNS anomalies may involve the kidney, heart, extremity, facial abnormalities, single umbilical artery, liver, and orthopedic malformations^7^.

Based on previous studies, PFA is usually associated with other structural anomalies, either CNS or extra-CNS. In general, isolated PFA of any type has better neurodevelopmental outcomes than cases with additional intracranial or extracranial abnormalities. MCM would have better neurodevelopmental outcomes than DWM, especially if isolated^8^.

In Saudi Arabia, we know little about the fetal and neonatal outcomes of PFA, although there is a high rate of genetic disease and consanguineous marriage. Ohaegbulam et al. found that the incidence of DMW in Tabuk city, Saudi Arabia was 1.24 for males and 0.78 for females, and it was associated with 3.5% of infantile hydrocephalus^9^.

This study aims to estimate the incidence and identify the different types of posterior fossa abnormalities in the King Abdulaziz Medical City (KAMC) population and assess the outcomes associated with different types of PFA, such as neurologic development, from 2017 to 2021.

## Results

**Figure 1:**
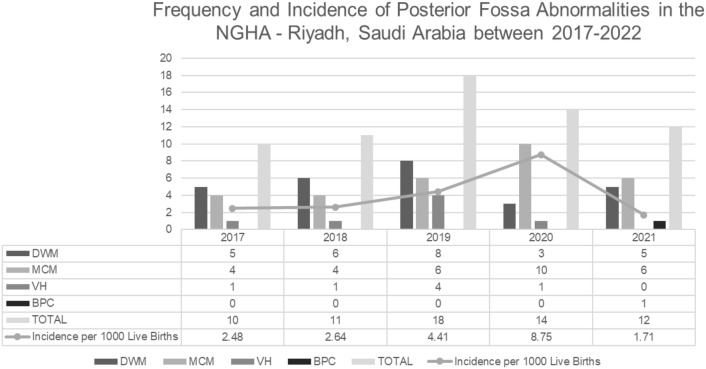
Frequency and Incidence* of Posterior Fossa Abnormalities in the KAMC—Riyadh, Saudi Arabia between 2017 and 2021. *Incidence was calculated as number of the posterior fossa abnormalities per total number of anatomy scans. *DWM* Dandy–Walker malformation, *MCM* mega cisterna magna, *BPC* Blake's pouch cyst, *VH* isolated vermian hypoplasia.

Sixty-five posterior fossa anomalies cases were identified in the KAMC—Riyadh, Saudi Arabia, between 2017 and 2021. The annual incidence rates were 2.48, 2.64, 4.41, 8.75, and 1.71 per 1000 anatomy scans for 2017, 2018, 2019, 2020, and 2021, respectively (Fig. 1). DWM and MCM were the most frequent PFA in this study. Between 2017 and 2019, the majority of the PFA were DWM, whereas MCM took over afterward during 2020 and 2021 (Fig. 1).

Over the 5 years, the cases of PFA were distributed as follows: 41.5% DWM, 46.2% MCM, 10.8% VH, and 1.5% BPC. The mean and standard deviation (SD) of maternal age was 31.6 ± 6.5 years. Approximately 14% of the mothers were primigravid. The prevalence of miscarriage was 38.5% among the mothers in this study. Approximately one-third of mothers in the study reported they were married consanguineously. Further, associated CNS abnormalities and extra CNS abnormalities were found in 52.3% and 41.5%, respectively, of the cases. A full description of the maternal and infant characteristics is included in Table 1.

**Table 1:** Maternal and infant characteristics of posterior fossa anomalies diagnosed in the KAMC—Riyadh, Saudi Arabia.

Variables		
Ultrasound finding of posterior fossa abnormalities, n (%)	DWM	27 (41.5)
	MCM	30 (46.2)
	VH	7 (10.8)
	BPC	1 (1.5)
Year of diagnosis, n (%)	2017	10 (15.4)
	2018	11 (16.9)
	2019	18 (27.7)
	2020	14 (21.5)
	2021	12 (18.5)
Maternal age, mean (SD)		31.6 (6.5)
Gravida, n (%)	1	9 (13.8)
	2	8 (12.3)
	3	16 (24.6)
	4	9 (13.8)
	5	11 (16.9)
	6 or more	5 (7.7)
Para, n (%)	0	9 (13.8)
	1	12 (18.5)
	2	19 (29.2)
	3	9 (13.8)
	4	10 (15.4)
	5 or more	6 (9.2)
Miscarriage, n (%)	0	40 (61.5)
	1	20 (30.8)
	2 or more	5 (7.7)
Live births, n (%)	0	11 (16.9)
	1	14 (21.5)
	2	14 (21.5)
	3	11 (16.9)
	4	9 (13.8)
	5 or more	6 (9.2)
Previous history of Congenital anomalies, n (%)	Yes	12 (18.5)
	No	53 (81.5)
Previous baby diagnosed with genetic disease, n (%)	Yes	4 (6.2)
	No	61 (93.8)
Family history of genetic disease, n (%)	Yes	3 (4.6)
	No	62 (95.4)
Consanguinity, n (%)	Yes	23 (35.4)
	No	18 (27.7)
	Undocumented	24 (36.9)
Maternal comorbidities, n (%)	Yes	12 (18.5)
	No	53 (81.5)
Gestational age at diagnosis in weeks, mean (SD)		28.2 (5.4)
Gestational age at delivery in weeks, mean (SD)		36.3 (4.8)
Gestational age at delivery, n (%)	Full term	39 (60.0)
	Pre-term	18 (27.7)
	Miscarriage	2 (3.1)
	Post-term	1 (1.5)
Ultrasound associated CNS abnormalities, n (%)	Yes	34 (52.3)
	No	31 (47.7)
Ultrasound extra CNS abnormalities, n (%)	Yes	27 (41.5)
	No	38 (58.5)
Mode of delivery, n (%)	Vaginal	39 (60.0)
	Cesarean section	21 (32.3)
	Undocumented	5 (7.7)
Onset Of labor, n (%)	Spontaneous vaginal delivery	21 (32.3)
	Induced vaginal delivery	23 (35.4)
	Elective cesarean section	16 (24.6)
	Undocumented	5 (7.7)
MRI result, n (%)	Normal	1 (1.5)
	Abnormal	4 (6.2)
	Undocumented	60 (92.3)
Result of Karyotype, n (%)	Normal	16 (24.6)
	Abnormal	5 (7.7)
	Undocumented	44 (67.7)
Fetal growth during pregnancy, n (%)	Appropriate for GA	51 (78.5)
	FGR	10 (15.4)
	Large	1 (1.5)
	Undocumented	3 (4.6)
Neonatal death, n (%)	Yes	4 (6.2)
	No	58 (89.2)
	Undocumented	3 (4.6)
Stillbirth, n (%)	Yes	9 (13.8)
	No	50 (76.9)
	Undocumented	6 (9.2)
NICU admission, n (%)	Yes	27 (41.5)
	No	26 (40)
	Undocumented	12 (18.5)
Dysmorphology, n (%)	Yes	23 (35.4)
	No	30 (46.2)
Weight (g), mean (SD)		2557.8 (820.8)
Head circumference (cm), mean (SD)		34.3 (3.4)
Head circumference, n (%)	Normal	34 (52.3)
	Macrocephaly	9 (13.8)
	Microcephaly	6 (9.2)
	Undocumented	16 (24.6)
Neurological exam, n (%)	Normal	24 (36.9)
	Abnormal	25 (38.5)
	Undocumented	16 (24.6)
Post-natal ultrasound, n (%)	Normal	7 (10.8)
	Abnormal	32 (49.2)
	Undocumented	26 (40)
Postnatal MRI, n (%)	Normal	1 (1.5)
	Abnormal	30 (46.2)
	Undocumented	34 (52.3)
Head and Neck examination, n (%)	Normal	24 (36.9)
	Abnormal	19 (29.2)
	Undocumented	22 (33.8)
Heart, n (%)	Normal	23 (35.4)
	Abnormal	15 (23.1)
	Undocumented	27 (41.5)
Kidneys, n (%)	Normal	17 (26.2)
	Abnormal	18 (27.7)
	Undocumented	30 (46.2)
Gastrointestinal, n (%)	Normal	26 (40)
	Abnormal	13 (20)
	Undocumented	26 (40)
WES, n (%)	Normal	6 (9.2)
	Abnormal	11 (16.9)
	Undocumented	48 (73.8)
WGS, n (%)	Normal	2 (3.1)
	Abnormal	2 (3.1)
	Undocumented	61 (93.8)
Postnatal karyotype, n (%)	Normal	21 (32.3)
	Abnormal	4 (6.2)
	Undocumented	40 (61.5)
CGH, n (%)	Normal	7 (10.8)
	Abnormal	7 (10.8)
	Undocumented	51 (78.5)
Infant death, n (%)	Yes	13 (20)
	No	16 (24.6)
	Undocumented	36 (55.4)
Death after 1 year, n (%)	Yes	6 (9.2)
	No	22 (33.8)
	Undocumented	37 (56.9)
Seizures, n (%)	Yes	14 (21.5)
	No	34 (52.3)
	Undocumented	17 (26.2)
Developmental/cognitive, n (%)	Normal	14 (21.5)
	Abnormal	21 (32.3)
	Undocumented	30 (46.2)
Neurosurgery intervention, n (%)	Yes	8 (12.3)
	No	41 (63.1)
	Undocumented	16 (24.6)
Feeding (oral/GT), n (%)	Oral	36 (55.4)
	NGT or OGT	5 (7.7)
	TPN	2 (3.1)
	GT	6 (9.2)
	Undocumented	16 (24.6)

*DWM* Dandy–Walker malformation, *MCM* mega cisterna magna, *BPC* Blake's pouch cyst, *VH* isolated vermian hypoplasia, *CNS* central nervous system, *MRI* magnetic resonance imaging, *FGR* fetal growth restriction, *NICU* neonatal intensive care unit, *WES* whole exome sequencing, *WGS* whole genome sequencing, *CGH* comparative genomic hybridization, *NGT* nasogastric tube, *OGT* orogastric tube, *TPN* total parenteral nutrition, *GT* gastrostomy tube.

Table 2 includes the maternal and infant characteristics of the two major posterior fossa anomalies diagnosed in the KAMC. Infants with DWM appear to have a higher proportion of associated CNS abnormalities than those without DWM (70.4% vs. 39.5%; p-value = 0.014). On the other hand, infants with MCM appear to have a significantly lower proportion of associated CNS abnormalities than those without MCM (36.7% vs. 65.7%; p-value = 0.019). Further, infants with DWM appear to have a higher proportion of dysmorphology than those without DWM (60.9% vs. 30%; p-value = 0.025). Further, compared to those without DWM, those with DWM had an abnormal neurological exam (70% vs. 37.9%; p-value = 0.027) and abnormal head and neck examination (63.2% vs. 29.2%; p-value = 0.026). Also, Infants with DWM appear to have a higher proportion of seizures than those without DWM (45% vs. 17.9%; p-value = 0.041). Finally, those with DWM were more likely to undergo neurosurgical intervention than those without DWM (30% vs. 6.9%; p-value = 0.049). On the other hand, those with MCM were not significantly different in the distribution of several maternal and infant characteristics from those without MCM (Table 2).

**Table 2:** Maternal and infant characteristics by the two major posterior fossa anomalies diagnosed in the KAMC—Riyadh, Saudi Arabia.

Variables		DWM			MCM		
		Yes	No	P-value	Yes	No	P-value
Maternal age, mean (SD)		31.9 (6.8)	31.4 (6.4)	0.766	32.7 (6.3)	30.7 (6.7)	0.224
Previous miscarriage, n (%)	Yes	8 (29.6)	17 (44.7)	0.217	14 (46.7)	11 (31.4)	0.208
	No	19 (70.4)	21 (55.3)		16 (53.3)	24 (68.6)	
Previous history of congenital anomalies, n (%)	Yes	7 (25.9)	5 (13.2)	0.191	5 (16.7)	7 (20.0)	0.730
	No	20 (74.1)	33 (86.8)		25 (83.3)	28 (80.0)	
Consanguinity, n (%)	Yes	11 (50.0)	7 (36.8)	0.397	6 (42.9)	12 (44.4)	0.923
	No	11 (50.0)	12 (63.2)		8 (57.1)	15 (55.6)	
Gestational age at diagnosis in weeks, mean (SD)		27.1 (6.5)	29.1 (4.4)	0.175	28.8 (4.0)	27.7 (6.4)	0.421
Gestational age at delivery in weeks, mean (SD)		35.3 (6.2)	37 (3.5)	0.224	36.4 (3.6)	36.2 (5.7)	0.852
Ultrasound associated CNS abnormalities, n (%)	Yes	19 (70.4)	15 (39.5)	0.014	11 (36.7)	23 (65.7)	0.019
	No	8 (29.6)	23 (60.5)		19 (63.3)	12 (34.3)	
Ultrasound extra CNS abnormalities, n (%)	Yes	14 (51.9)	13 (34.2)	0.155	11 (36.7)	16 (45.7)	0.461
	No	13 (48.1)	25 (65.8)		19 (63.3)	19 (54.3)	
Mode of delivery, n (%)	Vaginal	15 (57.7)	24 (70.6)	0.299	18 (69.2)	21 (61.8)	0.548
	CS	11 (42.3)	10 (29.4)		8 (30.8)	13 (38.2)	
NICU admission, n (%)	Yes	14 (60.9)	13 (43.3)	0.206	11 (50.0)	16 (51.6)	0.908
	No	9 (39.1)	17 (56.7)		11 (50.0)	15 (48.4)	
Dysmorphology, n (%)	Yes	14 (60.9)	9 (30.0)	0.025	7 (31.8)	16 (51.6)	0.152
	No	9 (39.1)	21 (70.0)		15 (68.2)	15 (48.4)	
Weight (g), mean (SD)		2451.3 (864.1)	2637.6 (791.1)	0.406	2538 (839.8)	2572.6 (819.4)	0.878
Head circumference (cm), mean (SD)		34.3 (3.6)	34.2 (3.4)	0.944	34.3 (3.9)	34.3 (3.1)	0.996
Head circumference, n (%)	Normal	12 (60.0)	22 (75.9)	0.236	14 (66.7)	20 (71.4)	0.720
	Abnormal	8 (40.0)	7 (24.1)		7 (33.3)	8 (28.6)	
Neurological exam, n (%)	Normal	6 (30.0)	18 (62.1)	0.027	13 (61.9)	11 (39.3)	0.117
	Abnormal	14 (70.0)	11 (37.9)		8 (38.1)	17 (60.7)	
Head and Neck dysmorphology, n (%)	Normal	7 (36.8)	17 (70.8)	0.026	10 (58.8)	14 (53.8)	0.748
	Abnormal	12 (63.2)	7 (29.2)		7 (41.2)	12 (46.2)	
Heart, n (%)	Normal	9 (52.9)	14 (66.7)	0.389	8 (61.5)	15 (60.0)	0.927
	Abnormal	8 (47.1)	7 (33.3)		5 (38.5)	10 (40.0)	
Kidneys, n (%)	Normal	10 (62.5)	7 (36.8)	0.130	4 (33.3)	13 (56.5)	0.289
	Abnormal	6 (37.5)	12 (63.2)		8 (66.7)	10 (43.5)	
Gastrointestinal , n (%)	Normal	11 (61.1)	15 (71.4)	0.496	10 (71.4)	16 (64.0)	0.733
	Abnormal	7 (38.9)	6 (28.6)		4 (28.6)	9 (36.0)	
Seizures, n (%)	Yes	9 (45.0)	5 (17.9)	0.041	4 (20.0)	10 (35.7)	0.338
	No	11 (55.0)	23 (82.1)		16 (80.0)	18 (64.3)	
Developmental/cognitive, n (%)	Normal	3 (21.4)	11 (52.4)	0.089	7 (50.0)	7 (33.3)	0.324
	Abnormal	11 (78.6)	10 (47.6)		7 (50.0)	14 (66.7)	
Neurosurgery intervention, n (%)	Yes	6 (30.0)	2 (6.9)	0.050	2 (9.5)	6 (21.4)	0.438
	No	14 (70.0)	27 (93.1)		19 (90.5)	22 (78.6)	

*DWM* Dandy–Walker malformation, *MCM* mega cisterna magna, *CS* cesarean section, *CNS* central nervous system, *NICU* neonatal intensive care unit.

A diagnosis of Dandy-Walker malformation was found in 27 pregnancies in total; CNS abnormalities were diagnosed in 20 cases, including absent cavum septum pellucidum in six fetuses and bilateral dilated lateral ventricles with absent cavum septum pellucidum in four, two fetuses were diagnosed with agenesis of the corpus callosum and eight with dilated lateral ventricles. Regarding the extra-CNS, a total of 10 pregnancies were identified, seven with cardiac anomalies and three with renal anomalies. Eleven fetuses diagnosed with MCM had associated CNS abnormalities: four with absent cavum septum pellucidum, four dilated lateral ventricles, two with agenesis of corpus callosum, and one with small cerebellum. Extra-CNS abnormalities in MCM were found in 11 cases, four with renal anomalies, including absent single kidney, polycystic kidney, and dilated renal pelvis. Three of MCM had hydrops fetalis. One was diagnosed with Down syndrome, the second one ended with stillbirth, and there was missing data for the third case. Cardiac anomalies were identified in three pregnancies with MCM and echogenic bowel in one case. VH was diagnosed in seven cases; three of them had associated CNS abnormalities, two with abnormal absent cavum septum pellucidum, one with dilated posterior fossa, and one diagnosed postnatally with Joubert syndrome. Only one of the VH cases had extra-CNS abnormalities, including atrial septal defect and dilated large bowel. Black's pouch case had dilated 4th ventricle and echogenic bowel. Dysmorphology was identified in 23 infants; (Table 3) shows the details of this dysmorphology and the associated PFA.

**Table 3:** Dysmorphological characteristics of infants with posterior fossa anomalies.

	Prenatal US diagnosis	Dysmorphological characteristics
Case 1	MCM	Macrocephaly, triangular face, high arched palate, sutures widely separated
Case 2	MCM	Low set ear, micrognathia, overlapped fingers, rocker bottom feet, undescended testis
Case 3	MCM	Microcephaly, broad prominent forhead, low set ears, retrognathia , hypotonia and clenched hands
Case 4	DWM	Rocker bottom feet
Case 5	MCM	Microcephaly, micrognathia, cleft palate, overlapping toes, single semin creases and camptodactyly
Case 6	DWM	Macrocephaly, frontal bossing, down slanted eyes, depressed nasal bridges, small pinched nose, microretrognathia, short neck and polydactyl in both hands
Case 7	MCM	Macrocephaly with wide anterior fontanel, depressed nasal bridge, down slanting palpebral fissure, hypertelorism, depressed nose, and low-set ears
Case 8	VH	Single simian crease in the left hand
Case 9	DWM	Low set small ears, microretrognothia, clenched hands, rocker bottom feet, short neck
Case 10	DWM	Microretrognathia, bitemporal narrowing, low set and posteriorly rotated ear, bulbar nose, short neck and dorsal edema of hands and foots
Case 11	DWM	Bilateral cleft palate, closed set eyes, dysmorphic ears, microcephaly
Case 12	DWM	Microcephaly, hypertolersim, depressed nasal bridge, Rocker bottom feet, clenched hand, narrow chest
Case 13	MCM	Microcephaly, hypertelorism, flat nasal root with wide nasal tip, puffy eyes, Prominent occipit, low set ear, short neck, clenched hands with overlapping fingers
Case 14	DWM	Microcephaly, depressed nasal bridge, low set ear, cleft palate, clenched hand, club foot
Case 15	DWM	Prominent forehead
Case 16	DWM	Brachycephalic with Prominent forehead, hypertelorism, down slanting palpebral fissures, low nasal bridge
Case 17	MCM	Trisomy 21 features: epicanthic folds, upslanting palpebral fissures, short neck, brachycephaly, broad forehead, hypertelorism and depressed nasal bridge
Case 18	DWM	Cleft lip and palate, microcephaly, hypertelorism, anophthalmia, lower limb deformity
Case 19	DWM	Microcephaly, cleft palate, closed set eye
Case 20	DWM	Micrognathia, right club foot
Case 21	DWM	Hypertelorism, low set ears, abnormal head shape
Case 22	DWM	Small eyes, prominent forehead, clenched hands
Case 23	VH	Depressed nasal bridge, almond shaped eyes and low set ears, right sided deviation of the mouth angle with intact nasolabial folds

*DWM* Dandy–Walker malformation, *MCM* mega cisterna magna, *BPC* Blake's pouch cyst, *VH* isolated vermian hypoplasia.

Fetal karyotype was performed in 21 cases, providing five abnormal karyotypes, two fetuses with MCM associated with CNS anomalies and three fetuses with DWM associated with CNS and non-CNS abnormalities. The abnormal karyotyping results were 4 trisomy 18 and 1 trisomy 13.

Genetic testing was done for 17 infants; 12 had abnormal results. Among the 12 patients, two infants had abnormal karyotypes, one had trisomy 18, and the other had a heterozygous likely pathogenic deletion within the cytogenic band 2q.23.1(147940954_148024399). The rest have a single gene mutation causing CNS abnormalities; four patients have pathogenic variants in different genes known to have CNS abnormalities, one patient with a pathogenic variant in the MPL gene, two affected infants with different variants in C5orf42 gene, the fourth patient has a pathogenic variant in ISPD gene (Table 4).

**Table 4:** Genetic investigations results in fetuses diagnosed with posterior fossa anomalies.

	Prenatal US diagnosis	Postnatal MRI	Genetic testing	Gene	variant	Class	Disease	Phenotype
Case 1	MCM associated with CNS abnormalities	Severe Supra and infratentorial cystic encephalomalacia with severe supratentorial ventriculomegaly and intraventricular hemorrhage	WES	JAM3	Homozygous variant c.406C > T p.(Gln136*) Both parents are heterozygous	3	Hemorrhagic destruction of the brain, subependymal calcification, and cataracts	1 day old boy, preterm 34 week, FGR, dysmorphic features with CHD (ASD, dysplastic bicuspid pulmonary valve) bilateral cystic encephalomalacia involving the cerebral hemisphere and cerebellum. There is diffuse ventriculomegaly with intraventricular hemorrhage
Case 2	MCM associated with extra CNS anomalies	–	WES	PDHA1	A heterozygous variant c.419-2A > G	2	X-linked pyruvate dehydrogenase E1-alpha deficiency	Neonate with multiple congenital anomalies including symmetrical FGR, cleft palate/Lip, ASD II, agenesis of corpus callosum, colpocephaly, and distal arthrogryposis associated with severe hypotonia, apnea and severe metabolic acidosis
Case 3	DWM	DWM	WES/WGS	MPL	Homozygous variant c.317C > T p.(Pro106Leu)	1	Congenital amegakaryocytic thrombocytopenia	41 months with bilateral preaxial polydactyly, Dandy-Walker malformation with VP shunt and thin corpus callosum
Case 4	DWM associated with Extra CNS anomalies	Holoprosencephaly	CGH array	Deletion of 83 kb in 2q.23.1	Deletion of 83 kb in 2q.23.1(147940954_148024399) × 1, this region include 5′UTR of the ORC4 gene mutation in the MBD5 gene may cause neurodevelopmental and psychiatric trait	Likely pathogenic	middle Interhemispheric holoprosencephaly with no midline structural anomalies	28 month days old baby girl, Twin B, case of middle interhemispheric holoprosencephaly with no midline structural anomalies, dysmorphic features, developmental delay and no family history of similar finding but mother had GDM during pregnancy
Case 5	MCM associated with CNS and Extra CNS anomalies	–	WES	TCIRG1	Homozygous variant in TCIRG1 gene c.1630G > A p.(Val544Met) in exon 14	3	An autosomal recessive osteopetrosis type 1	Dysmorphic features, brain ventriculomegaly and osteopetrosis
				MTMR2	Homozygous variant in MTMR2 gene c.25_26del p.(Leu10Trpfs*50) in exon 1	3	Charcot-Marie-Tooth disease type 4B1, an autosomal recessive disorder	
Case 6	DWM associated with extra CNS anomalies	–	Karyotype	47,XY, + 18	Chromosome analysis revealed: 47,XY, + 18	Pathogenic	Edward syndrome with CHD	A 24 days boy with CHD
Case 7	DWM	1.Simplification of the cerebral gyral pattern along with thickening of the cortex in keeping with migration anomaly. 2.Diffuse brain edema and hypomyelination. 3. Periventricular white matter loss with severe thinning of the corpus callosum. 4. Severe cerebellar hyperplasia along with mildly thin brainstem and abnormal signal intensity in the pons. Marked engorgement of the cortical veins	WES	PNPLA8	A variant of uncertain significance in the PNPLA8 gene was identified in a homozygous state c.1690G > C p.(Ala564Pro) exon 9	3	Simplification of the cerebral gyral pattern along with thickening of the cortex in keeping with migration anomaly Diffuse brain edema and hypomyelination Periventricular white matter loss with severe thinning of the corpus callosum Severe cerebellar hypoplasia along with mildly thin brainstem and abnormal signal intensity in the pons	An infant with Simplification of the cerebral gyral pattern, thinning of the corpus callosum, and Severe cerebellar hypoplasia, with positive family history of brain anomalies to rule out underlying genetic disorders
Case 8	DWM	Bilateral frontal polymicrogyria with no other intracranial findings suggestive of underlying syndromic aetiology. The large posterior fossa cyst likely represent Blake's pouch cyst. No hydrocephalous	WES	COL18A1	A variant in COL18A1 Gene c.5071_5072del p.(Trp1691Alafs*16)	2	Knobloch syndrome	5 years old female with nystagmus, macrocephaly, hypotonia. Bilateral frontal polymicrogyria, Dandy–Walker cyst, vermis hypoplasia and abnormal whiter signals. Also she has high myopia, staphyloma and tilted disc
Case 9	DWM associated with CNS and extra CNS anomalies	Microcephaly with simplified gyral pattern and pontocerebellar hypoplasia. The findings could be related to neurodegenerative disease, syndromic association or remote antenatal insult (e.g. congenital infection)	WES	PNPLA8	c.1748_1749del p.(Tyr583Trpfs*17)	2	An autosomal recessive PNPLA8-related phenotype	4 months old female has microcephaly, cleft palate, Pontocerebellar hypoplasia, ASD II, Neonatal onset of epileptic encephalopathy and hypotonia Muscle biopsy confirmed myopathy with mitochondrial abnormalities
Case 10	DWM associated with extra CNS anomalies	Findings consistent with Joubert syndrome	WES	C5orf42	c.7778G > A p.(Trp2593*) Karyotype normal	1	Joubert syndrome type 17	4 years old male with global developmental delay and seizures Brain MRI showed posterior fossa with markedly hypoplastic vermis, abnormally thickened and horizontally oriented superior cerebellar peduncles with molar tooth configuration of the midbrain. Prominent cisterna magna. Abnormally oriented cerebellar hemispheres
Case 11	DWM associated CNS anomalies	The findings are highly suggestive of Walker–Warburg syndrome with supratentorial hydrocephalus	WES	ISPD	Homozygote pathogenic variant in ISPD gene c.1186G > T p.(Glu396*)	1	AR congenital muscular dystrophy dystroglycanopathy	7 months old baby girl 136 days, on Comfort care, he is case of:Ÿ Ÿ Preterm 36 + 5 , product of Elective C-section Walker–Warburg syndrome confirmed by molecular test (with supratentorial hydrocephalus, Lissencephaly, muscular dystrophy, retinal dysplasia)
Case12	VH associated with CNS anomalies	Findings of Joubert syndrome in the posterior fossa. Small posterior fossa subdural hemorrhage	WES	C5orf42	Homozygous pathogenic variant in C5orf42 gene c.7988_7989 (p.Gly2663Alafs*40)	1	Joubert Syndrome type 17	A 3 and half months old boy with confirmed Joubert Syndrome. He doesn’t have polydactyly or renal disease. He has normal eye examination

*DWM* Dandy–Walker malformation, *MCM* mega cisterna magna, *BPC* Blake's pouch cyst, *VH* isolated vermian hypoplasia, *CNS* central nervous system, *MRI* magnetic resonance imaging, *FGR* fetal growth restriction, *CHD* congenital heart disease, *ASD* atrial septal defect, *VP* ventriculoperitoneal, *GDM* gestational diabetes mellitus, *NICU* neonatal intensive care unit, *WES* whole exome sequencing, *WGS* whole genome sequencing, *CGH* comparative genomic hybridization.

We also identified four patients with likely pathogenic variants in the following genes PDHA1, PNPLA8, JAM3and in COL18A1 genes causing CNS manifestations. Also, one patient has a different variant of uncertain significance in the PNPLA8 gene that suggests its rule for pathogenicity. Finally, we found two variants in two different genes in an infant with ventriculomegaly osteopetrosis (Table 4).

## Discussion

Our study’s findings showed a single-tertiary center experience and indicated that DWM and MCM were the most common PFA, consistent with other research^3^. Incidence rates per 1000 anatomy scans were 2.48, 2.64, 4.41, 8.75, and 1.71 for 2017, 2018, 2019, 2020, and 2021, respectively. DWM is a rare disease with an estimated incidence of 1 per 30,000 births^10,11^. A common CNS finding in DWM cases is ventriculomegaly^3^, which might need a ventriculoperitoneal (VP) shunt in severe cases. A previous study found that 62.7% of DWM cases required VP shunts; however, our findings showed fewer patients needed VP shunts, 30%^12^. The majority of DWM cases in our study have been associated with CNS and extra CNS anomalies. DWM infants, therefore, have a high rate of seizure occurrence. Previous neurosurgical series indicate that the combination of DWM and hydrocephalus is associated with abnormal neurologic development in 40–70% of survivors^13,14^. Our findings showed a slightly higher percentage, with 78.6% of DWM cases diagnosed postnatally as developmental delay; Hydrocephaly was developed in only six cases. However, hydrocephalus was reported as the most frequent CNS complication in DWM, and Venkatesan et al. results showed there is a high risk of requiring a ventriculoperitoneal shunt and developing epilepsy during early childhood^15–17^. In addition, the results of a previous systematic review demonstrate an increased risk of abnormal neurodevelopmental outcomes in children with a prenatal diagnosis of isolated DWM^18^.

In general, the reported neurodevelopmental outcome of isolated MCM is favorable^18^, similar to our study of seven cases of isolated MCM with a normal neurodevelopmental outcome. However, concurrent CNS abnormalities were related to a worse prognosis in children with MCM^8^; in our study, all cases of MCM associated with CNS abnormalities were diagnosed later as a developmental delay, except one patient was diagnosed with cytomegalovirus (CMV) infection.

The precise diagnosis of isolated BPC is not always simple and is not confirmed prenatally in some cases^3,19^. Our result showed one case of BPC was diagnosed prenatally but was not confirmed postnatally as the diagnosis was DWM with no ventriculomegaly by postnatal MRI. The existence of extra CNS anomalies was correlated with a worse prognosis compared to the absence of such abnormalities^5^. CNS-associated abnormalities were 52% in cases of DWM and MCM, which is consistent with a recent study's finding that CNS was the most frequent ultrasound abnormality^20^. The most frequently observed extra CNS anomalies were renal and congenital heart disease in 27.7% and 23.1% of all PFA cases, respectively, which is similar to the previous study's result^20^. Our result is consistent with the previous report, which showed the most frequent extra CNS anomalies were infantile polycystic kidneys in DWM infants^21^. In addition, eight cases with congenital heart disease were found to have developmental delays. According to Seker et al., cardiac anomalies play a crucial impact in infant prognosis^22^. There have been reports of concomitant dysmorphology similar to our study's findings, including deformities of the face and a cleft palate and lip^20,23,24^.

Genetic and chromosomal abnormalities are important factors in DMW etiology^25^. Although a normal karyotype can coexist with DWM, the 3rd, 9th, 13th, and 18th chromosomes are most frequently linked to concurrent chromosomal abnormalities^26^. In our study, genetic testing was performed on 17 patients, of which 12 had abnormal results. Total (fetal and postnatal) karyotype was performed in 23 cases, of which six had abnormal results. We have found 12 infants with abnormal results, two with abnormal karyotypes, and the remaining ten with gene mutations. Among the patients with abnormal karyotypes, we found four cases with trisomy 18 and one with trisomy 13, known in previous studies to be associated with posterior fossa anomalies^25^. The other case has microdeletion in the cytogenetic band 2q23.1, which was not known to be associated with posterior fossa abnormalities^27^. From this study, we cannot conclude if the PFA are a coincidence or an expanding phenotype for this microdeletion. Further study is needed to clarify this association. The remaining ten infants have a single gene mutation, including the MPL gene, which causes rare inherited bone marrow failure syndrome, which was reported in one case report to be associated with hypoplastic cerebellar vermis with a communication between the fourth ventricle and the cisterna magna^28^. Two affected infants with different variants in the C5orf42 gene caused Joubert syndrome type 17, which was not specifically associated with posterior fossa anomalies. However, Joubert syndrome was reported in some cases with PFA, including DWM^29^. The fourth patient has a pathogenic variant in the ISPD gene causing Muscular dystrophy-dystroglycanopathy, which is known to be associated with DWM^30^. We also identified four patients with likely pathogenic variants, including PDHA1 causing Pyruvate dehydrogenase E1-alpha deficiency, JAM3 causing hemorrhagic destruction of the brain, subependymal calcification, and congenital cataracts (HDBSCC) with the neonatal onset and COL18A1 gene causing Knobloch syndrome, type 1. All genes are known to be associated with CNS manifestations; however, none of them is reported to be associated with PFA^31–33^. In addition, two patients were found to have a PNPLA8 gene mutation, which causes mitochondrial myopathy with lactic acidosis, and it was not reported previously with PFA^34^. Finally, we have identified one infant with two variants in two different genes; one gene is MTMR2, causing Charcot-Marie-Tooth disease, type 4B1, and the other is aTCIRG1, causing osteopetrosis, autosomal recessive 1^35,36^. The latter has been reported in one case of severe autosomal recessive osteopetrosis associated with Dandy–Walker syndrome and agenesis of the corpus callosum^37^.

Non-genetic contributor to PFA is infection^38^. According to certain reports, PFA may be related to CMV infection^39,40^. Intracranial calcification is the most frequent abnormality observed in infants with congenital CMV infection on neuroradiologic imaging, in addition to other intracranial abnormalities that include ventriculomegaly, white matter abnormalities, neuronal migration abnormalities, and extensive destructive encephalopathy^40^. We reported one case of congenital CMV in which prenatal ultrasound showed mega cisterna magna associated with right ventriculomegaly.

The consanguineous marriage rate is 51% in Saudi Arabia^41^. According to our findings, one-third of the parents of fetuses with PFA were consanguineous, and 61% of these fetuses have abnormal genetic testing**.** Our results show that half (7 out of 13) of the genetic testing is an autosomal recessive disease which is supported by a previous study that demonstrates autosomal recessive pathogenic mutation is the major cause of genetic disease in Saudi patients^42^. In addition, previous data showed a high proportion of DWM in consanguineous marriages, reaching 44%^21^.

To our knowledge, this is the only study of fetal posterior fossa anomalies in Saudi Arabia. Although it is limited because it is retrospective, with missing data and a small sample size, it provides information on posterior fossa anomalies in our population and the association of genetic disease in consanguineous marriage. MRI criteria for diagnosing DWM were classic since it is a retrospective study from 2017 to 2021, and this is one of the limitations. The criteria that most accurately characterize the modern DWM phenotype are an enlarged tegmentovermian angle, an obtuse fastigial recess, an unpaired caudal lobule (tail sign), inferior, predominant VH, and inferolateral displacement of the tela choroidea/choroid plexus^43^.

## Conclusion

The most common PFA in the KAMC population is DWM and MCM. Developmental delay is more likely when there are associated CNS anomalies. The autosomal recessive pathogenic mutation is the major cause of genetic disease in Saudi patients diagnosed with PFA, and the possibility of identifying new phenotypic associations is high. Therefore, preconception and genetic counseling programs should be implemented for the consanguineous parents.

## Methods

This was a retrospective, single-center cohort study conducted between January 2017 and December 2021 in King Abdulaziz Medical City (KAMC), Riyadh, Saudi Arabia. KAMC is one of the largest tertiary centers in the region, with 1973 beds. The Department of Obstetrics and Gynecology performs about 8000 deliveries each year, with mother and fetal outcomes similar to international standards^44^. Initially, cases were identified from the perinatal review meeting list held weekly to discuss the new fetal cases in the clinic. All fetuses diagnosed with PFA were included. The diagnosis was made according to transabdominal ultrasound mid-trimester examination images using the standard axial planes for screening exams, including trans ventricular, trans thalamic, and trans cerebellar planes^45^, which were reviewed and confirmed by a fetal-medicine consultant. A fetal MRI was ordered when diagnostics certainty was required. The MRI criteria for diagnosing DWM include lack of the lower part of the vermis, anterior rotation, upward displacement, and hypoplasia of the remaining vermis. The fastigium's angle may be fattened or absent. There is a large bossing posterior fossa with torcular elevation, and normal or hypoplastic cerebellar hemispheres are displaced anterolaterally^46^. The data were obtained from the medical records; all maternal and infant information was collected from BESTcare, the hospital’s electronic health system. The extracted maternal data included maternal age, gravidity, parity, gestational age at diagnosis, gestational age at delivery, family history of genetic disease, fetal death, stillbirth, and consanguineous marriage. Fetal records were reviewed for birth weight, neonatal intensive care unit (NICU) admission, neonatal death, neuroimaging, dysmorphology, extra-CNS organ involvement, seizure, developmental and cognitive outcome, neurosurgery intervention, and genetic testing, including whole genome sequencing WGS, whole exome sequencing WES and chromosomal analysis.

Posterior fossa abnormalities were defined based on a proposed morphological approach, including:

1. Dandy–Walker malformation (DWM) was defined by the classic triad of enlargement of the posterior fossa with an elevated cerebellar tentorium, complete or partial agenesis of the cerebellar vermis, and dilatation of the fourth ventricle.
2. Mega cisterna magna (MCM) was defined as enlargement of the cisterna magna > 10 mm with normal cerebellar vermis.
3. Blake’s pouch cyst (BPC) was defined as the presence of an upwardly displaced normal cerebellar vermis, normal appearance of the fastigium, tentorium, and size of the cisterna magna.
4. Isolated vermian hypoplasia (VH) was defined as a normally formed vermis of smaller size, and the posterior fossa is otherwise of normal size and anatomy^47^.

Marriage between two blood-related individuals with a common ancestor, like a second cousin or closer relative, is considered consanguineous marriage^48^. No formal evaluation of neurodevelopment was conducted. However, the outcome was deemed normal if the child had normal routine developmental screenings.

### Research ethics approval

The study was approved by the institutional review board of King Abdullah International Medical Research Center (KAIMARC) (IRB/1833/21). The research was conducted in compliance with the relevant regulations and rules. In accordance with the hospital policy, informed consent was obtained from all individuals and/or their legal guardian(s) for all genetic testing.

### Data analysis

Data were analyzed using SPSS version 21.0 software (SPSS Inc., Chicago, IL, USA). Descriptive statistics were reported as frequencies (n) and percentages (%) for categorical/nominal variables and mean and standard deviation (SD) for continuous variables. In addition, the annual incidence of posterior fossa anomalies was calculated as the annual number of cases divided by the total number of anatomy scans in the corresponding year.

Since the two major posterior fossa anomalies diagnosed in the KAMC were DWM and MCM, we aimed to assess their relationships and several maternal and infant characteristics. Thus, we used the chi-square test, Fisher exact test when required, to explore the relationship between each type of the anomalies and several maternal and infant characteristics measured on a categorical scale. We also used independent samples t-test, Mann–Whitney where applicable, to compare the means of other maternal and infant characteristics measured in interval scale across DWM and MCM anomalies.

## Data Availability

The data supporting this study's findings are available from the corresponding author upon reasonable request.

## References

[1] Milani HJF, Barreto EQDS, Araujo E (2019). Ultrasonographic evaluation of the fetal central nervous system: Review of guidelines. Radiol. Bras..

[2] Niesen CE (2002). Malformations of the posterior fossa: Current perspectives. Proc. Semin. Pediatr. Neurol..

[3] D’Antonio F, Khalil A, Garel C (2016). Systematic review and metaanalysis of isolated posterior fossa malformations on prenatal ultrasound imaging (part 1): Nomenclature, diagnostic accuracy and associated anomalies. Ultrasound Obstet. Gynecol..

[4] Parisi MA, Dobyns WB (2003). Human malformations of the midbrain and hindbrain: Review and proposed classification scheme. Mol. Genet. Metab..

[5] Patek KJ, Kline-Fath BM, Hopkin RJ (2012). Posterior fossa anomalies diagnosed with fetal MRI: Associated anomalies and neurodevelopmental outcomes. Prenat. Diagn..

[6] Ecker JL, Shipp TD, Bromley B (2000). The sonographic diagnosis of Dandy–Walker and Dandy–Walker variant: Associated findings and outcomes. Prenat. Diagn..

[7] Long A, Moran P, Robson S (2006). Outcome of fetal cerebral posterior fossa anomalies. Prenat. Diagn..

[8] Bolduc ME, Limperopoulos C (2009). Neurodevelopmental outcomes in children with cerebellar malformations: A systematic review. Dev. Med. Child Neurol..

[9] Ohaegbulam SC, Afifi H (2001). Dandy–Walker syndrome: Incidence in a defined population of Tabuk, Saudi Arabia. Neuroepidemiology.

[10] Pilu G, Perolo A, Falco P (2000). Ultrasound of the fetal central nervous system. Curr. Opin. Obstet. Gynecol..

[11] Altman NR, Naidich TP, Braffman BH (1992). Posterior fossa malformations. Am. J. Neuroradiol..

[12] D’Antonio F, Khalil A, Garel C (2016). Systematic review and meta-analysis of isolated posterior fossa malformations on prenatal imaging (part 2): Neurodevelopmental outcome. Ultrasound. Obstet. Gynecol..

[13] Hirsch JF, Pierre-Kahn A, Renier D (1984). The Dandy–Walker malformation: A review of 40 cases. J. Neurosurg..

[14] Sawaya R, McLaurin RL (1981). Dandy–Walker syndrome. Clinical analysis of 23 cases. J. Neurosurg..

[15] Ecker JL, Shipp TD, Bromley B, Benacerraf B (2000). The sonographic diagnosis of Dandy–Walker and Dandy–Walker variant: Associated findings and outcomes. Prenat. Diagn..

[16] Di Nora A, Costanza G, Pizzo F, Di Mari A, Sapuppo A, Basile A, Fiumara A, Pavone P (2023). Dandy–Walker malformation and variants: Clinical features and associated anomalies in 28 affected children-a single retrospective study and a review of the literature. Acta Neurol. Belg..

[17] Venkatesan C, Kline-Fath B, Horn PS, Poisson KE, Hopkin R, Nagaraj UD (2021). Short- and long-term outcomes of prenatally diagnosed Dandy–Walker malformation, vermian hypoplasia, and blake pouch cyst. J. Child Neurol..

[18] D’Antonio F, Khalil A, Garel C (2016). Systematic review and meta-analysis of isolated posterior fossa malformations on prenatal imaging (part 2): Neurodevelopmental outcome. Ultrasound Obstet. Gynecol..

[19] Kau T, Marterer R, Kottke R (2020). Blakeʼs Pouch Cysts and differential diagnoses in prenatal and postnatal MRI. Clin. Neuroradiol..

[20] Sun Y, Wang T, Zhang N, Zhang P, Li Y (2023). Clinical features and genetic analysis of Dandy–Walker syndrome. BMC Pregnancy Childbirth..

[21] Has R, Ermiş H, Yüksel A (2004). Dandy–Walker malformation: A review of 78 cases diagnosed by prenatal sonography. Fetal Diagn. Ther..

[22] Şeker E, Aslan B, Aydın E (2022). Long term outcomes of fetal posterior fossa abnormalities diagnosed with fetal MRI. J. Turk. Ger. Gynecol. Assoc..

[23] Guibaud L, Larroque A, Ville D, Sanlaville D, Till M, Gaucherand P, Pracros JP, des Portes, V. (2012). Prenatal diagnosis of 'isolated' Dandy–Walker malformation: Imaging findings and prenatal counselling. Prenat. Diagn..

[24] Aydın E, Turgal M, Can S, Özyüncü Ö (2016). Posterior fossa anomalies: Related anomalies and the methods of pregnancy termination. Perinat. J..

[25] Murray JC, Johnson JA, Bird TD (1985). Dandy–Walker malformation: Etiologic heterogeneity and empiric recurrence risks. Clin. Genet..

[26] Imataka G, Yamanouchi H, Arisaka O (2007). Dandy–Walker syndrome and chromosomal abnormalities. Congenit. Anom. (Kyoto)..

[27] Van Bon BW, Koolen DA, Brueton L (2010). The 2q23.1 microdeletion syndrome: Clinical and behavioural phenotype. Eur. J. Hum. Genet..

[28] Ihara K, Ishii E, Eguchi M (1999). Identification of mutations in the c-mpl gene in congenital amegakaryocytic thrombocytopenia. Proc. Natl. Acad. Sci. U. S. A..

[29] Stambolliu E, Ioakeim-Ioannidou M, Kontokostas K (2017). The most common comorbidities in Dandy–Walker syndrome patients: A systematic review of case reports. J. Child Neurol..

[30] Online Mendelian Inheritance in Man, OMIM^®^. Johns Hopkins University, Baltimore, MD. MIM Number: {614631}: {12/14/2020}: https://www.omim.org/entry/616052#references (accessed 20 Jan 2023).

[31] Online Mendelian Inheritance in Man, OMIM^®^. Johns Hopkins University, Baltimore, MD. MIM Number: {300502}: {01/13/2021}. https://www.omim.org/entry/312170 (accessed 20 Jan 2023).

[32] Online Mendelian Inheritance in Man, OMIM^®^. Johns Hopkins University, Baltimore, MD. MIM Number: {606871}: {02/11/2014}. https://www.omim.org/entry/613730. (accessed 20 Jan 2023).

[33] Online Mendelian Inheritance in Man, OMIM^®^. Johns Hopkins University, Baltimore, MD. MIM Number: {120328}: {07/21/2022}. World Wide Web URL:. https://www.omim.org/entry/267750 (accessed 20 Jan 2023).

[34] Online Mendelian Inheritance in Man, OMIM^®^. Johns Hopkins University, Baltimore, MD. MIM Number: {612123}: {04/21/2017}. World Wide Web URL. https://www.omim.org/entry/251950 (accessed 20 Jan 2023).

[35] Online Mendelian Inheritance in Man, OMIM^®^. Johns Hopkins University, Baltimore, MD. MIM Number: {603557}: {06/30/2009}. World Wide Web URL. https://www.omim.org/entry/601382 (accessed 20 Jan 2023).

[36] Online Mendelian Inheritance in Man, OMIM^®^. Johns Hopkins University, Baltimore, MD. MIM Number: {604592}: {07/13/2018}. World Wide Web URL: https://www.omim.org/entry/259700 (accessed 20 Jan 2023).

[37] Ben Hamouda H, Sfar MN, Braham R (2001). Association of severe autosomal recessive osteopetrosis and Dandy–Walker syndrome with agenesis of the corpus callosum. Acta Orthop. Belg..

[38] Bosemani T, Orman G, Boltshauser E (2015). Congenital abnormalities of the posterior fossa. Radiographics.

[39] Dogan Y, Yuksel A, Kalelioglu IH (2011). Intracranial ultrasound abnormalities and fetal cytomegalovirus infection: Report of 8 cases and review of the literature. Fetal Diagn. Ther..

[40] Puvabanditsin S, Dumitrescu C, Garrow E (2008). Not a Dandy–Walker malformation but congenital cytomegalovirus infection. HK J. Paediatr..

[41] Husain MA, Bunyan MA (1997). Consanguineous marriages in a Saudi population and the effect of inbreeding on prenatal and postnatal mortality. Ann. Trop. Paediatr..

[42] Monies D, Abouelhoda M, AlSayed M (2017). The landscape of genetic diseases in Saudi Arabia based on the first 1000 diagnostic panels and exomes. Hum. Genet..

[43] Whitehead MT, Barkovich MJ, Sidpra J, Alves CA, Mirsky DM, Öztekin Ö, Bhattacharya D, Lucato LT, Sudhakar S, Taranath A, Andronikou S, Prabhu SP, Aldinger KA, Haldipur P, Millen KJ, Barkovich AJ, Boltshauser E, Dobyns WB, Mankad K (2022). Refining the neuroimaging definition of the Dandy–Walker phenotype. AJNR Am. J. Neuroradiol..

[44] Madkhali AM, Al Ghamdi SO, Al-Sum H (2021). Framework for obstetrics and gynecology department change management in response to COVID 19 pandemic: A tertiary center experience. Ann. Thorac. Med..

[45] Salomon LJ, Alfirevic Z, Berghella V (2011). Practice guidelines for performance of the routine mid-trimester fetal ultrasound scan. Ultrasound Obstet. Gynecol..

[46] Klein O, Pierre-Kahn A, Boddaert N, Parisot D, Brunelle F (2003). Dandy–Walker malformation: Prenatal diagnosis and prognosis. Childs Nerv. Syst..

[47] Tortori-Donati P, Fondelli MP, Rossi A (1996). Cystic malformations of the posterior cranial fossa originating from a defect of the posterior membranous area. Mega cisterna magna and persisting Blake’s pouch: Two separate entities. Childs Nerv. Syst..

[48] Zakzouk S, El-Sayed Y, Bafaqeeh SA (1993). Consanguinity and hereditary hearing impairment among Saudi population. Ann. Saudi Med..

